# Impact of preanalytical conditions on plasma concentration and size distribution of extracellular vesicles using Nanoparticle Tracking Analysis

**DOI:** 10.1038/s41598-018-35401-8

**Published:** 2018-11-21

**Authors:** Simin Jamaly, Cathrine Ramberg, Randi Olsen, Nadezhda Latysheva, Paul Webster, Timofey Sovershaev, Sigrid K. Brækkan, John-Bjarne Hansen

**Affiliations:** 1 K.G. Jebsen - Thrombosis Research and Expertise Center (TREC), Department of Clinical Medicine, UiT - The Arctic University of Norway, Tromsø, Norway; 2 Division of Internal Medicine, University Hospital of North Norway, Tromsø, Norway; 3 Department of Electron Microscopy, Institute of Medical Biology, Faculty of Health Sciences, UiT- The Arctic University of Norway, Tromsø, Norway; 4 Center for Electron Microscopy and Microanalysis, Oak Crest Institute of Science, Monrovia, California, United States of America

## Abstract

Optimal pre-analytical handling is essential for valid measurements of plasma concentration and size distribution of extracellular vesicles (EVs). We investigated the impact of plasma preparation, various anticoagulants (Citrate, EDTA, CTAD, Heparin), and fasting status on concentration and size distribution of EVs measured by Nanoparticle Tracking Analysis (NTA). Blood was drawn from 10 healthy volunteers to investigate the impact of plasma preparation and anticoagulants, and from 40 individuals from a population-based study to investigate the impact of postprandial lipidemia. Plasma concentration of EVs was measured by NTA after isolation by high-speed centrifugation, and size distribution of EVs was determined using NTA and scanning electron microscopy (SEM). Plasma concentrations and size distributions of EVs were essentially similar for the various anticoagulants. Transmission electron microscopy (TEM) confirmed the presence of EVs. TEM and SEM-analyses showed that the EVs retained spherical morphology after high-speed centrifugation. Plasma EVs were not changed in postprandial lipidemia, but the mean sizes of VLDL particles were increased and interfered with EV measurements (explained 66% of the variation in EVs-concentration in the postprandial phase). Optimization of procedures for separating VLDL particles and EVs is therefore needed before NTA-assessment of EVs can be used as biomarkers of disease.

## Introduction

Extracellular vesicles (EVs), including exosomes (30–100 nm in diameter) and microvesicles (100–1000 nm in diameter), are bilayer membrane vesicles released from various cells into their surroundings^1^. The EVs are characterized by their size, mechanism of cellular release, and their content of proteins, RNA, and DNA molecules derived from the parental cell^2^. The functional properties of EVs are dependent on the cellular origin and pathological conditions^3,4^. Elevated plasma levels of EVs, and microvesicles in particular, have been associated with several disease states such as atherosclerosis^5,6^, diabetes^7^, cancer^8,9^, arterial cardiovascular diseases^10–12^ and venous thromboembolism^13,14^. Although the majority of EVs in plasma have diameters below 200 nm^15,16^, most studies relating plasma levels of EVs with diseases have been carried out using flow cytometry for EV measurements with lower detection limits above 200 nm. Therefore, it would be attractive to use Nanoparticle Tracking Analysis (NTA) to determine plasma concentration of EVs in the size range of 50–1000 nm^15,17^ in order to assess associations between the predominantly smaller EVs and disease states.

Measurement of plasma EVs by NTA could possibly provide novel information on the role of EVs as potential biomarkers of risk, diagnosis, and prognosis of various diseases. However, the clinical application is currently hampered by methodological concerns related to NTA assessment of EVs. In human plasma, more than 98% of the particles detected by NTA are very low density lipoprotein (VLDL) particles^15,18^, and the measured particle concentrations are associated with the triglyceride concentration both under fasting and postprandial conditions^19^. Isolation of EVs from plasma is therefore an essential pre-analytical step before NTA measurement. However, isolation of EVs by differential centrifugation may affect EV morphology, promote aggregation of EVs, and to some extent pellet lipoproteins^20–22^. In clinical and epidemiological studies, plasma is most often prepared by single centrifugation at 1500–3000 × g for 10–20 min, yielding platelet poor plasma (PPP), before storage in biobanks. High-speed centrifugation to achieve platelet free plasma (PFP) from PPP prior to freezing has been shown to lower the concentration of platelet-derived EVs compared to plasma subjected only to lower speed centrifugation (PPP) before freezing^23^. However, it is not known whether high-speed centrifugation after thawing of PPP would affect concentration and size distribution of plasma derived EVs compared to PFP prepared by double centrifugation (including one low and one high-speed centrifugation step) before freezing. Furthermore, previous studies comparing the impact of various anticoagulants on plasma concentrations of EVs, assessed by flow cytometry, have shown higher levels of EVs in heparin than in sodium citrate, acid-citrate dextrose (ACD) or sodium citrate theophylline adenosine dipyridamole (CTAD)^24,25^.

Pre-analytical conditions such as centrifugation steps, choice of anticoagulant, and fasting status may impact the plasma concentration and size distribution of EVs determined by NTA. We therefore aimed to investigate the impact of plasma preparation, assessed by freezing plasma before (PPP) or after (PFP) a second high-speed centrifugation, various anticoagulants in commercial blood collection tubes (Citrate, EDTA, CTAD, and Heparin), and fasting status on plasma concentration and size distribution of EVs using NTA and SEM.

## Material and Methods

### Study participants

Ten healthy volunteers (5 men and 5 women, aged 28–55 years) were recruited from the research staff, and they donated blood used to investigate the method of blood collection, centrifugation steps for plasma preparation and EV isolation, and choice of anticoagulant on the concentration and size distribution of plasma EVs. The study was approved by the regional ethical committee (REK Nord), and was conducted in accordance with relevant guidelines and regulations. Informed written consent was obtained from all participants.

Forty healthy subjects, 20 to 80 years of age, were recruited from a general population-based study (the Tromsø study) in order to investigate whether fasting and postprandial lipoproteins would affect the concentration and size distribution of plasma EVs. They underwent a screening visit including a complete medical history, physical examination, a self-administrated questionnaire which also included dietary habits, physical exercise, and alcohol consumption, and blood samples were taken with special emphasis on exclusion criteria. Exclusion criteria were any of the following conditions: regular use of lipid-lowering drugs (statins, resins or nicotinic acid derivates), estrogen supplementation or oral anticoagulants, cancer or other serious life-threatening medical conditions, present or previous cardiovascular diseases, recurrent venous thrombosis, diabetes mellitus, hypothyroidism, renal, hepatic, or psychiatric disease, and current abuse of alcohol or drugs. Informed written consent was obtained from all participants, and the Regional Committee for Research Ethics approved the study. The study was performed at the Clinical Research Unit at the University Hospital of North-Norway.

### Blood collection

Blood was drawn from an antecubital vein using a 21 Gauge needle in the morning (08:30 am). Tourniquet was only used to find a vein and was opened after needle insertion. Blood was drawn into regular commercially available blood collection tubes (BD Vacutainers) (BD Bioscience, New Jersey, US) with the following anticoagulants; Sodium citrate (2.7 ml, REF363048), Sodium heparin (6.0 ml, REF367876), Ethylenediaminetetraacetic acid (K2E-EDTA) (6.0 ml, REF367864) and buffered Sodium Citrate Theophylline Adenosine Dipyridamole (CTAD) (4.5 ml, REF367599). The first few millilitres of blood were drawn into a dummy tube that was discarded afterwards. The blood collection tubes were gently inverted several times in order to mix anticoagulants with blood. The blood collection tubes were not transported, as blood collection and plasma preparation was performed in the same laboratory. The blood collection tubes were held in upright racks until centrifuged at room temperature. Blood cell counts were performed at baseline using Micros60 (ABX Diagnostics, Montpellier, France).

### Plasma and EV Preparation

Platelet poor plasma (PPP) was prepared from anticoagulated whole blood by centrifugation at 3000 × g for 10 minutes at room temperature within 30 minutes after blood collection. PPP was then either subjected to a second high-speed centrifugation at 13,500 × g for 2 minutes to achieve platelet free plasma (PFP) or aliquoted and stored at −70 °C for at least 48 hours. After thawing, PPP was then centrifuged at 13,500 × g for 2 minutes to get rid of platelets and cell debris. PFP was then diluted with Hanks/Hepes buffer (130 mM NaCl, 5.4 mM KCl, 1.3 mM CaCl_2_, 0.8 mM MgSO_4_, 0.44 mM Na_2_HPO_4_, 20 mM HEPES, pH 7.4) (1:10 dilution) (to be used for electron microscopy) or sterile filtered Dulbecco’s phosphate buffered saline (DPBS) without CaCl_2_ and MgCl_2_ (Sigma-Aldrich, St. Luis, MO, USA) (1:20 dilution) (to be used for EV concentration and size distribution)^26^. EVs were pelleted from PFP by centrifugation at 20,000 g for 30 minutes at room temperature (Beckman OptimaTM LE-80K Ultracentrifuge, swinging bucket rotor SW 40 TI). The supernatant was discarded and the EV pellet re-suspended in DPBS, snap frozen^27^ and stored at −70 °C until further analysis. For electron microscopy the EV pellet was re-suspended in double filtered Hanks/Hepes buffer and fixed in 4% formaldehyde until further analysis.

### Nanoparticle Tracking Analysis (NTA)

EV concentration and size distribution were determined using NanoSight NS300 (Malvern Instruments Ltd., Worcestershire, UK) equipped with a 488 nm blue laser and a CMOS camera. EV samples were thawed in 37 °C water immediately prior to analysis and diluted (50–100×) in DPBS. Samples were manually introduced to the instrument using a syringe. Samples were captured at ambient temperature with automatic temperature monitoring. Three separate dilutions of the samples were used for analysis. Each dilution was captured 3 times 60 seconds (camera level 16), and the sample was refreshed between captures. The gasket was cleaned between each sample. The nine resulting videos were analysed with NTA software version 3.0 (detection threshold 5). Mean values for concentration and size distribution were calculated.

### Transmission Electron Microscopy (TEM)

TEM was performed on isolated EVs. The EV pellets were re-suspended in 50 µl of Hanks/Hepes buffer and fixed in 4% formaldehyde in 200 ml Hepes overnight.

### TEM of Ultrathin sections

To improve localization of EVs for TEM, the EVs were adsorbed onto an epoxy resin substrate containing colloidal gold particles. The substrate was prepared by adsorbing 15 nm gold particles (Department cell biology, University of Utrecht, the Netherlands) on formvar/carbon coated copper specimen grids and then embedded the gold-coated grids in a thin layer of epoxy resin between two layers of Aclar film and polymerized at 60 °C for 48 hours. The EV suspension was placed on the gold-loaded, epoxy-embedded specimen grids and in 1% glutaraldehyde, postfixated in 1% OsO_4_ and stained with 1% aqueous uranyl acetate. The EVs on the epoxy-embedded grids were dehydrated in a graded series of ethanol, infiltrated in an Epon Equivalent (AGAR 100, DDSA, MNA and DMP-30, Agar Scientific, UK) and polymerized at 60 °C for 48 hrs. Ultrathin sections of the embedded EVs were prepared using Ultracut S ultramicrotome (Lieca Microsystems, Vienna, Austria) and a Diatome diamond knife (Diatome, Biel, Switzerland). Images using a JEOL JEM 1010 transmission electron microscope (Tokyo, Japan) were acquired with a Morada camera system (Olympus Soft Imageing System, Münster, Germany).

### Immune electron microscopy

EV’s were fixed with 1% buffered glutaraldehyde and adsorbed onto carbon-formvar coated specimen grids before immunolabelling. In short, unspecific labelling was blocked on 0.1% cold water fish skin gelatin (CWFSG) (Sigma G-7765) and 1.5% bovine serum albumin. Samples were incubated with anti-annexin V (Anx5) antibody (abcam, Cat# ab14196), diluted in Anx5 binding buffer (BD Pharmingen, Cat#556454) and protein A-gold (University of Utrecht, The Netherlands). All immunoreagents were diluted in CWFSG and the grids washed in PBS between each step. The grids were finally fixed in 1% glutaraldehyde, washed in distilled water and dried in 1.8% Methylcellulose containing 0.3% uranyl acetate.

### Scanning Electron Microscopy (SEM)

To obtain an overview of the morphology and measure particle size of EV samples, isolated EVs were prepared for SEM analysis. To study the surface size, shape, and features of EVs, they were negatively stained on formvar/carbon coated copper grids. Grids floated on sample drops for 30 minutes were treated with 1% glutaraldehyde, washed in PBS and ddH2O and contrasted/dried with the addition of 1.8% methyl cellulose and 0.3% uranyl acetate according to Tokuyasu^28,29^. EV size measurements were performed in the iTEM program (Olympus Soft Imaging Solutions, Münster, Germany) by measuring shortest diameter of at least 200 EV on SEM pictures. The start and end of every diameter was set manually and the diameter was calculated by the program. Grid were mounted on specimen holder and coated with gold/palladium before examination in the SEM. The images were obtained using a Zeizz Merlin VP compact scanning microscope.

### Fat tolerance test

A fat-tolerance test was conducted using a test meal prepared from standard porridge cream containing 70% of calories from fat of which 66% saturated fat, 32% monounsaturated fat and 2% polyunsaturated fat. The test meals were served with two teaspoons of sugar, cinnamon, and two glasses (150 ml each) of sugar-free juice. The test meals were freshly prepared each morning. A weight-adjusted meal (1 gram fat per kg body weight) was served at 8:00 a.m. and consumed over a 15-minutes period. The participants were allowed to drink 350 ml calorie-free beverages and eat an apple during the following 8 hrs. Blood was drawn from an antecubital vein in the morning at 7:45 a.m., after a 12 hour overnight fast and a 48 hour refrain of exhaustive physical exercise and alcohol consumption, and then 2, 4, 6, and 8 hours after the meal, using a 19-gauge needle in a vacutainer system with minimal stasis for serum and plasma preparations. Blood for plasma preparation was collected into 4.5 ml vacutainers (Becton Dickinson, Meylan Cedex, France) containing 0.129 M sodium citrate (1 vol anticoagulant and 9 vol whole blood) or EDTA (K_3_ – EDTA 40 µl, 0.37 mol/L per tube) as anticoagulant. Serum was prepared by clotting whole blood in a glass tube at room temperature for 1 hour. Serum and plasma was prepared by centrifugation at 2000 g for 15 minutes at 22 °C, transferred into cryovials (Greiner laboratechnik, Nürtringen, Germany) in aliquots of 1 ml and stored at −70 °C until further analysis.

Serum lipids were analyzed on an ABX Pentra 400 (Horiba ABX Diagnostics, Montpellier, France) with reagents from Horiba ABX Diagnostics (Montpellier, France). Proton nuclear magnetic resonance (NMR) spectroscopy was used to determine mean particle sizes of the main lipoprotein classes (very-low-density lipoprotein (VLDL), low-density-lipoprotein (LDL), and high-density lipoprotein (HDL)) along with concentrations of 10 lipoprotein subclasses (chylomicron/large VLDL, medium VLDL, small VLDL, intermediate-density lipoprotein (IDL), large LDL, small LDL also reported as medium small LDL and very small LDL, large HDL, medium HDL, and small HDL) in fasting and 4-hours postprandial citrated plasma at LipoScience Inc., Railegh, NC, USA. Plasma concentrations of EVs in fasting and 4-hours postprandial citrated plasmas were determined using NTA as previously described.

### Statistics

Median values and interquartile ranges for continuous data (EV concentrations) were presented as data was not normally distributed. To test for differences in EV concentrations between anticoagulants, we used Friedman’s test for non-parametric and dependent continuous data. Bar graphs were used to display (i) EV concentrations according to size categories of EVs (<100 nm, 100–199 nm, 200–299 nm, 300–1000 nm), and (ii) mean sizes of EVs measured by NTA and SEM in the different anticoagulants. The correlation between triglycerides and EV concentrations, as well as VLDL and EV concentrations, was calculated using Pearson correlation coefficient. All analyses were performed using IBM SPSS Statistics version 22 (Armonk, NY, USA).

## Results

The baseline characteristics of the ten healthy volunteers (aged 28–55 years) recruited from the research staff and the forty subjects (aged 28–76 years) recruited from the general population health study are shown in Table 1.

**Table 1:** Characteristics of the volunteers recruited from the research staff (n = 10) and the participants of the cohort study (n = 40).

	Volunteers (n = 10)	Cohort (n = 40)
Women, n (%)	5 (50)	20 (50)
Age (years)	41 ± 9	56 ± 14
Body mass index (kg/m^2^)		28.0 ± 4.0
Haemoglobin (g/dL)	13.9 ± 1.2	14.4 ± 1.3
Leukocytes (10^9^/L)	5.1 ± 0.9	6.3 ± 1.7
Platelet count (10^9^/L)	229 ± 47	249 ± 62
Total cholesterol (mmol/L)		5.69 ± 1.35
HDL cholesterol (mmol/L)		1.32 ± 0.45
Triglycerides (mmol/L)		1.33 ± 0.82

Values are means ± standard deviations or numbers with percentage in brackets.

**Figure 1:**
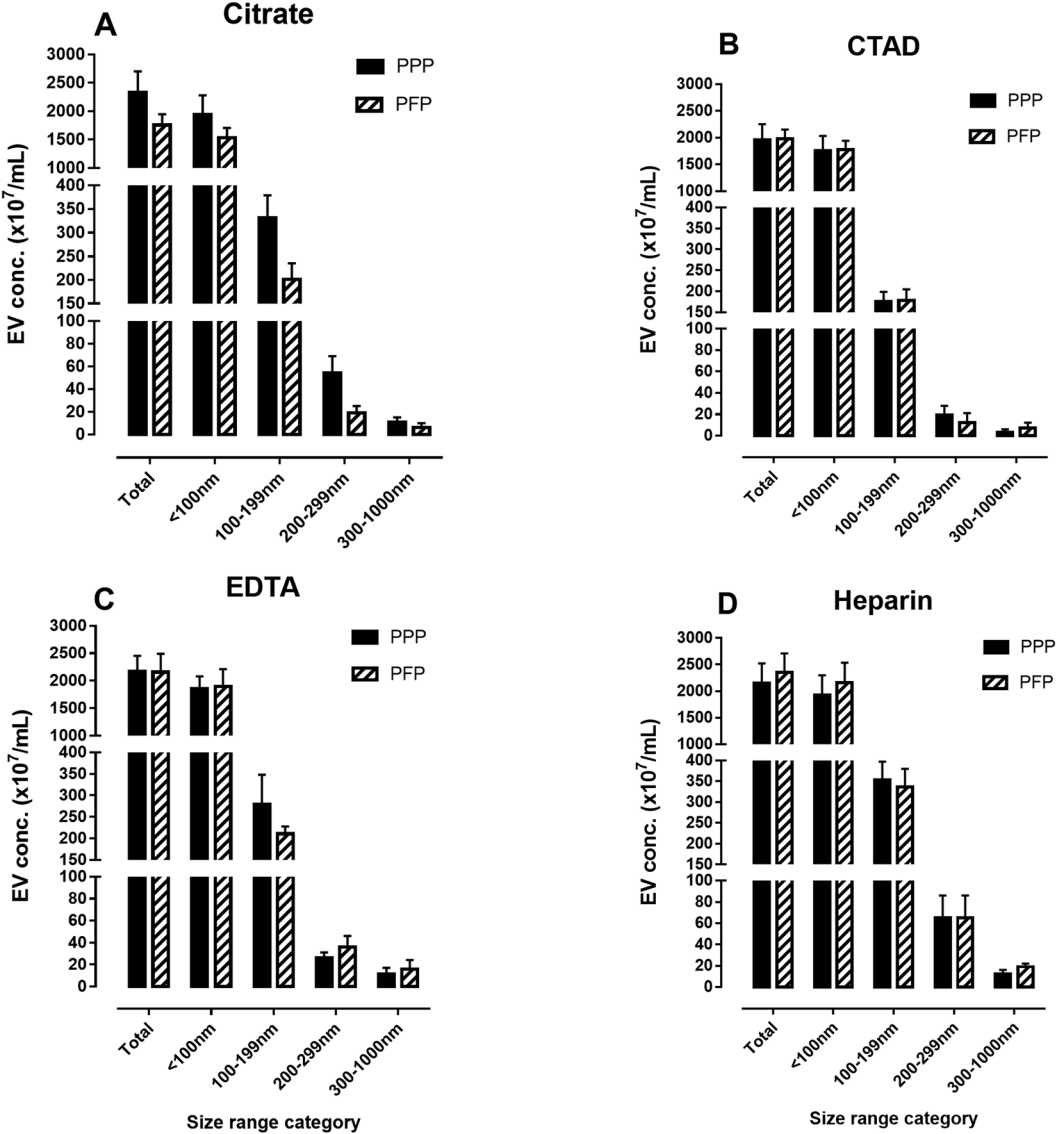
Panels of bar graphs showing concentrations across size categories of plasma EVs frozen before (platelet poor plasma, PPP) and after (platelet free plasma, PFP) high-speed centrifugation in order to get rid of cell debris and platelets. After thawing, plasmas frozen as PPP were subjected to high-speed centrifugation before isolation of plasma EVs. The panels represent blood anticoagulated with citrate (panel A), CTAD (panel B), EDTA (panel C), and heparin (panel D). EV concentrations were measured by nanoparticle tracking analysis (NTA).Values are means with standard error of the means (SEM) (n = 10 in each group). EDTA: Ethylenediaminetetraacetic acid, CTAD: sodium citrate theophylline adenosine dipyridamole.

The impact of freezing PPP compared to PFP on concentration and size distribution of plasma-derived EVs is shown in Fig. 1, panel A–D. There were no statistical differences in total concentration and size distribution of EVs between plasmas prepared as PPP and PFP before freezing in any of the anticoagulants used (Fig. 1).

The impact of the four different anticoagulants on concentration and size distribution of plasma EVs are displayed in Table 2. Neither the total plasma concentration, nor concentrations of EVs within defined size categories (<100 nm, 100–199 nm, 200–299 nm and 300–1000 nm) differed significantly between the anticoagulants. However, although not statistically significant, blood anticoagulated with sodium citrate showed the lowest plasma concentration of EVs across all size categories, whereas heparinized plasma yielded an almost 2-fold higher concentration of large EVs (300–1000 nm) compared to the other anticoagulants (Table 2). The majority of EVs were in the smaller size categories (<100 nm: 76.4% to 78.2%, 100–199 nm: 18.0% to 20.2%, 200–299 nm: 2.7% to 3.5%, 300–1000 nm: 0.7% to 1.3%) and the size distribution did not differ between anticoagulants (Table 2).

**Table 2:** Plasma concentration (10^7^ per mL) of extracellular vesicles (EVs) according to type of anticoagulant and size categories of EV.Values are expressed as median values with interquartile ranges (n = 10).

EV size category	Citrate	EDTA	CTAD	Heparin	*p*
<100 nm	1133 (1066–2034)	1514 (1074–2039)	1420 (928–1926)	1314 (928–2207)	0.1
100–199 nm	262 (200–450)	402 (270–443)	319 (265–474)	313 (287–372)	0.2
200–299 nm	41 (19–71)	53 (38–65)	63 (40–105)	54 (35–76)	0.7
300–1000 nm	13 (2–20)	14 (6–19)	13 (5–36)	22 (9–52)	0.5
Total Concentration	1588 (1331–2403)	2017 (1510–2528)	1980 (1320–2514)	1658 (1342–2700)	0.2

Values are expressed as medians and interquartile ranges, nm: nanometer, EV: Extracellular vesicle, EDTA: Ethylenediaminetetraacetic acid, CTAD: sodium citrate theophylline adenosine dipyridamole.

**Figure 2:**
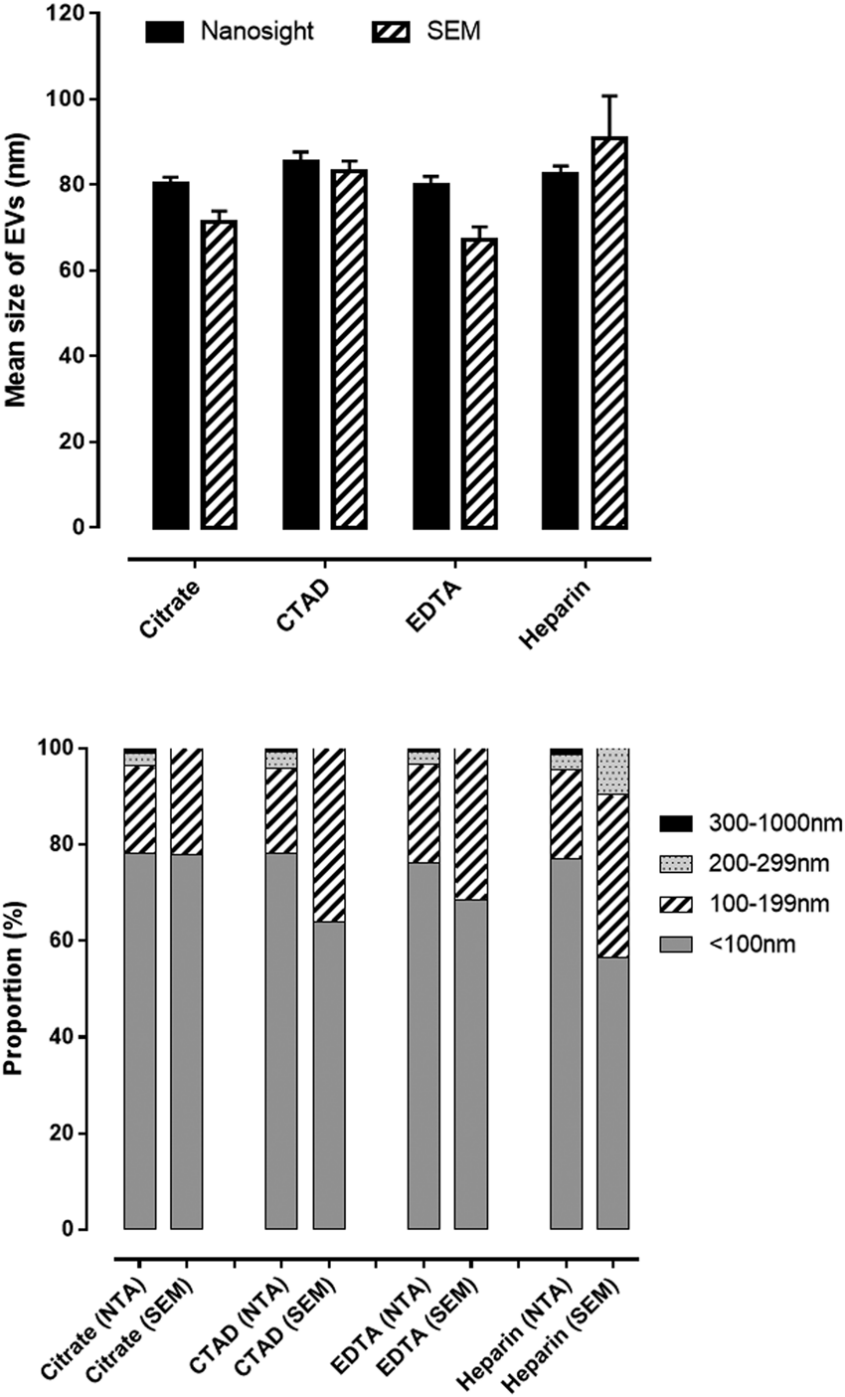
Bar graphs displaying mean sizes of plasma EVs measured by nanoparticle tracking analysis (NTA) and scanning electron microscopy (SEM) (upper panel) and size distribution (lower panel) across categories of various blood anticoagulants (upper panel). Values are means with standard error of the means (upper panel) or proportions (lower panel). EDTA: Ethylenediaminetetraacetic acid, CTAD: sodium citrate theophylline adenosine dipyridamole.

The mean size of the EVs was marginally lower (Fig. 2, upper panel) and the size distribution was shifted towards smaller EVs (<199 nm) when EVs were sized by SEM compared to NTA (Fig. 2, lower panel). Similar to NTA, the majority of EVs determined by SEM were below 200 nm in diameter and large vesicles were rarely seen.

**Figure 3:**
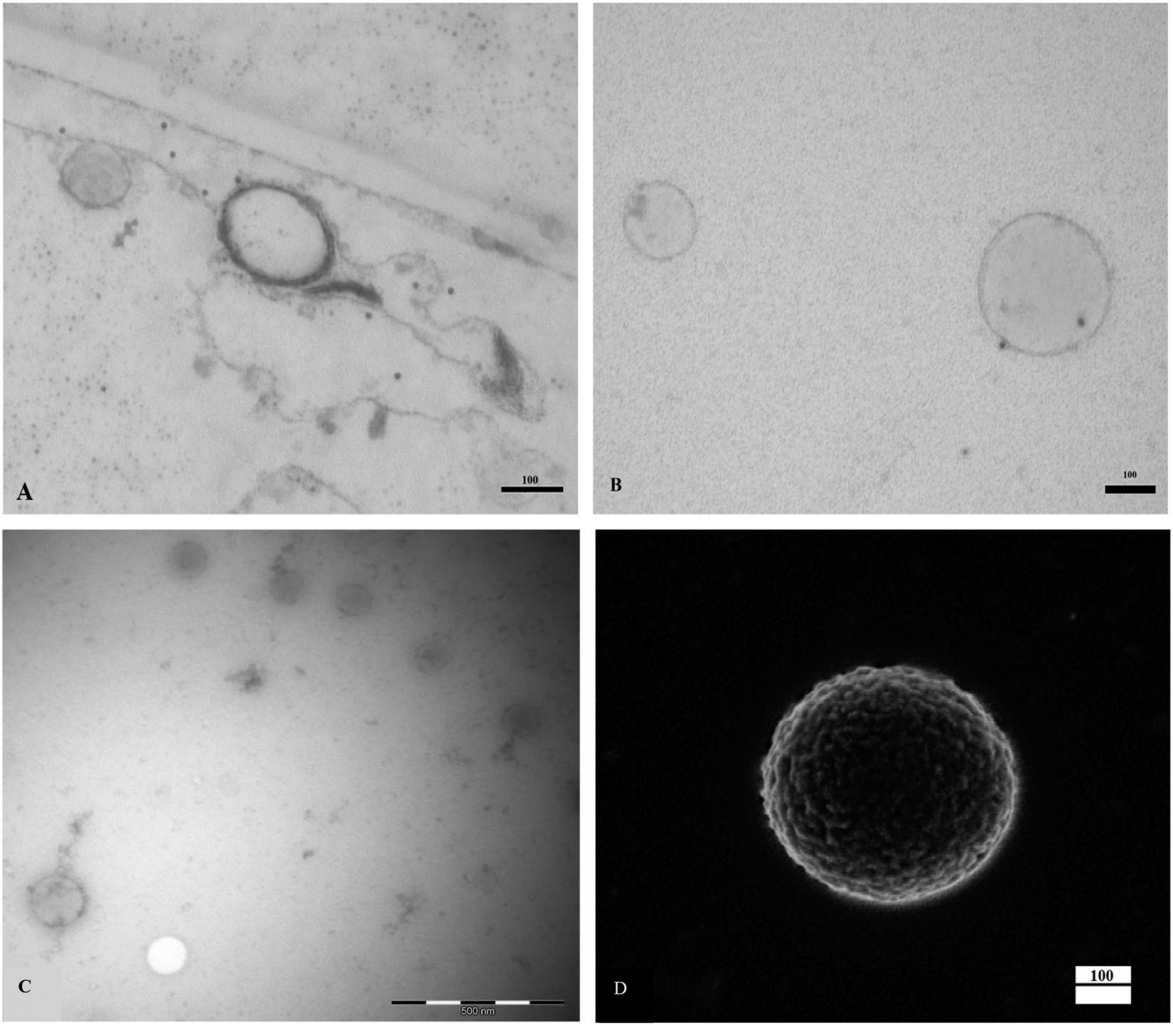
Visualization of Extracellular Vesicles (EVs) by scanning electron microscopy (SEM) and transmission electron microscopy (TEM). (Panel A) EVs with phospholipid bilayer membrane after ultra-thin sectioning of the Epon blocks. (Panel B) Representative transmission electron micrograph of purified, negatively stained annexin positive and negative bilayer membrane extracellular vesicles. (Panel C) Representative transmission electron micrograph of plasma purified, negatively stained EVs. (Panel D) Representative micrograph of an EV by SEM, isolated from EDTA peripheral blood of a healthy donor at the working distance of 2.6 mm and an accelerating voltage of 2.00 kV (Original magnification 61.12 KX). EVs were spherical and presented uneven non-smooth surfaces.

TEM was applied to confirm the presence of EVs after isolation by high-speed centrifugation from plasma samples. TEM of negatively stained (Fig. 3A) and ultrathin sections (Fig. 3B) revealed that the majority of isolated vesicles were EVs, characterized by the bilayer phospholipid membrane. Even though concern has been raised about EV aggregation and morphological changes as a result of high-speed centrifugation, we could not identify EV aggregates, nor large variations in the EV morphologies, as the main proportion of vesicles identified were spherical (Fig. 3C) and presented uneven non-smooth surfaces (Fig. 3D). Lipoprotein vesicles, characterized by the lack of a bilayer surface membrane, were rarely seen.

**Figure 4:**
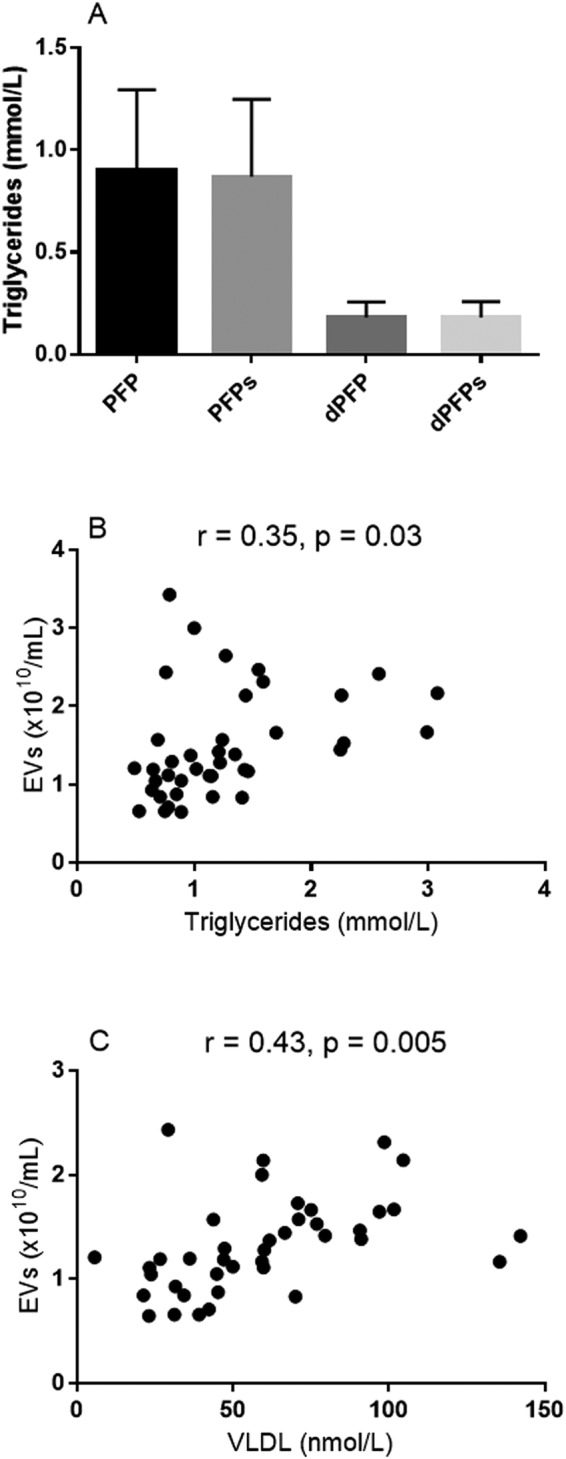
Bar graphs showing concentration of triglycerides before and after high-speed centrifugation (20,000 × g for 30 minutes) in undiluted (PFP) and diluted (dPFP, diluted 1:5 in DPBS) samples (panel A, bar graphs are means with 1 standard deviation), and dot-plots showing correlations between plasma concentration of EVs and serum triglycerides (panel B) and plasma VLDL particles (panel C) under fasting conditions. Proton nuclear magnetic resonance (NMR) spectroscopy was used to determine mean particle sizes of the main lipoprotein classes, and EV concentration was measured by NTA.

In order to determine if the concentration of triglycerides would change, and lipoproteins would sediment with EVs after a high speed centrifugation (20,000 × g for 30 minutes), we measured the levels of plasma lipoproteins before and after centrifugation, in both diluted and undiluted plasma. We found that the plasma concentration of triglycerides was equal before and after high-speed centrifugation for both undiluted and diluted (1:5 in DPBS) plasma (Fig. 4, panel A). Despite the minimal sedimentation of VLDL particles by high-speed centrifugation due to unchanged plasma concentration of triglycerides, we found moderate correlations between EVs and plasma concentrations of triglycerides (Fig. 4, panel B) and VLDL particles (Fig. 4, panel C) under fasting conditions. Concentrations of serum triglycerides and VLDL particles explained (r^2^) 13% and 19%, respectively, of the plasma variation of EVs.

**Figure 5:**
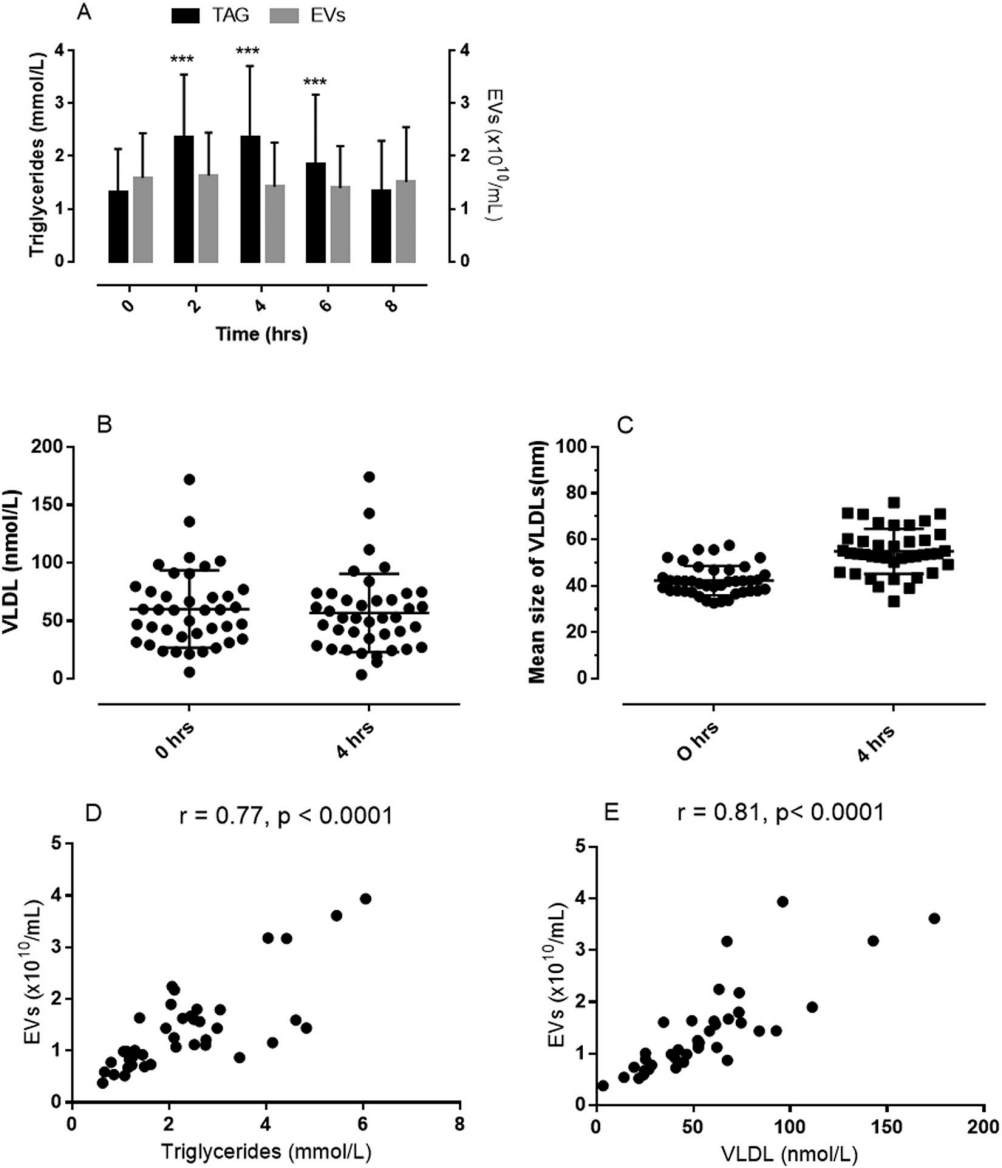
Bar graphs showing concentrations of serum triglycerides and plasma EVs before (fasting) and every second hour after ingestion of a standardized high fat meal (panel A, bar graphs are means with 1 standard deviation, ***p < 0.0001 from fasting levels). Dot plots showing median and 25 to 75% percentiles of plasma concentration of VLDL particles (panel B) and mean sizes of VLDL particles (panel C); and dot plots showing correlations between plasma concentration of EVs (measured by NTA) and serum triglycerides (panel D) and plasma VLDL particles (panel E) 4 hours after ingestion of a standardized high fat meal.

Ingestion of a standardized high-fat meal (1 g/kg body weight) was accompanied by a significant increase in serum triglycerides, which peaked at 4 hours, and returned to baseline levels within 8 hours after the meal (Fig. 5A). The postprandial lipidemia was not accompanied by significant changes in the concentration of EVs in plasma determined by NTA (Fig. 5A). Similarly, the plasma concentration of VLDL particles did not change from the fasting to the postprandial state (Fig. 5B), but the median diameter of VLDL particles increased from 42 ± 6 nm in fasting plasma to 55 ± 9 nm in postprandial plasma (p < 0.0001) (Fig. 5C). However, serum triglycerides and the concentration of VLDL particles in plasma collected 4 hours after ingestion of the meal showed a strong correlation with the plasma concentration of EVs and explained 59–66% of the variation in plasma EVs (r^2^ = 0.59 and 0.66, respectively) (Fig. 5D and E).

## Discussion

We aimed to investigate the impact of various preanalytical conditions such as centrifugation steps for plasma preparation and EV isolation, choice of anticoagulant, and fasting status on plasma concentration and size distribution of EVs determined by NTA. The size distribution of plasma EVs was similar when determined by NTA and SEM, and the majority of EVs were round-shaped. The plasma concentration and size distribution of EVs were essentially similar in plasmas isolated from blood with various anticoagulants, even though the plasma concentration of large EVs was almost 2-fold higher in heparinized plasma compared to the other anticoagulants. A bilayer membrane of EVs was demonstrated in the majority of particles in the EV pellet by two separate TEM methods. The plasma concentration and size distribution of EVs, isolated from plasma by high-speed centrifugation and determined by NTA, were similar in blood collected before and 4 hours after ingestion of a high-fat meal. Even though triglyceride levels remained unchanged after high-speed centrifugation, serum concentrations of triglycerides and plasma concentrations of VLDL particles correlated with the concentration of EVs, particularly in the postprandial state.

Isolation of EVs from plasma by high-speed centrifugation may elicit shape change, aggregates and/or damage of EVs. In our study, the mean diameter of EVs isolated from plasma by high-speed centrifugation was 80–90 nm. Our results were very similar to those reported by Dragovic *et al*. who measured vesicles directly in plasma after labelling with a specific cell tracker dye^17^. We used two independent methods (NTA and SEM) to determine the size distribution of the EV fraction, and the results were similar for the two methods. NTA assessment of the size distribution revealed that only 3.5–5% of the EVs had a diameter above 200 nm, and that only 0.7–1.3% of the EVs had a diameter above 300 nm. The low proportion of EVs with a diameter above 2–300 nm, which is the lower detection limit of conventional flow cytometers, may explain the huge differences in observed plasma concentrations of EVs measured by flow cytometry and NTA.

Previous studies comparing the impact of various anticoagulants on plasma concentrations of EVs assessed by flow cytometry, have shown higher levels of EVs in heparin than in sodium citrate, ACD or CTAD as anticoagulants^24,25^. In line with these studies, we found that the plasma level of large EVs (>300 nm), which is detectable by flow cytometry, was 2-fold higher in plasma anticoagulated by heparin compared to plasma containing the other anticoagulants. In our study, blood anticoagulated with EDTA, a stronger chelator than citrate, displayed highest plasma concentrations of EVs, supporting the notion that EDTA promotes formation of artefactual microvesicles due to platelet activation^30^. Citrated plasma displayed the lowest median concentrations of EVs across all size categories. Citrate plays a role in membrane phospholipid remodelling and may thereby partially inhibit vesiculation^30^. Our findings of a lower EV concentration in samples with citrate indicates that *in vitro* vesiculation may occur to a larger extent with the other anticoagulants, and support the recommended use of citrate as anticoagulant for studies on EVs.

Previous studies using Nanoparticle Tracking Analysis of PFP have reported a particle concentration of approximately 1.5 × 10^12^/mL in plasma^17^. However, NTA does not allow for distinction between EVs and other particles within the same size range in plasma. Triglyceride-rich lipoproteins (chylomicrons and very-low-density lipoproteins) are in molar excess, but of similar size to cell-derived EVs^18,31,32^, and can be detected in light scatter. Previous studies showed that the apparent plasma concentration of EVs measured by NTA was highly correlated to the plasma concentration of triglycerides^19^, and that the concentration of EVs declined by more than 98% when only vesicles labelled with a cell tracker dye were counted^17^. To avoid interference of triglyceride-rich lipoproteins on the NTA measurements of EVs, we isolated EVs from plasma by high-speed centrifugation and re-suspended the pellet in a particle-free buffer. In our study, the median concentrations of EVs isolated from plasma varied between 1.6–2.0 × 10^10^/mL with an interquartile range from 1.3 to 2.7 × 10^10^/mL, which is in line with the results based on specific labelling of EVs by a cell tracker dye^17^.

Our findings provide several lines of evidence for at least partial separation of EVs from triglyceride-rich lipoproteins by high-speed centrifugation of plasma. First, the concentration of triglycerides in the plasma supernatant remained unchanged after high-speed centrifugation, suggesting that VLDL-particles in fasting blood samples were not pelleted and mainly remained floating. Second, even though serum triglycerides and the concentration of VLDL particles in plasma from fasting individuals displayed a moderate correlation with the plasma concentration of EVs, it only explained 13–19% of the variation in plasma EVs, suggesting that the particle count, measured by NTA, was not dominated by VLDL particles. Third, we developed a novel procedure to section the EVs, and could thereby clearly visualize the bilayer phospholipid membrane which distinguishes EVs from lipoproteins. Fourth, the results of our TEM analysis confirmed that the majority of isolated vesicles in our samples were in fact EVs (characterized by the bilayer phospholipid membrane) and not lipoproteins or protein complexes.

Ingestion of a standardized high-fat meal was accompanied by significant increase in serum triglycerides which peaked at 4 hours and returned to baseline levels 8 hours after the meal without affecting the plasma levels of EVs determined by NTA. However, serum triglycerides and the concentration of VLDL particles in plasma collected 4 hours after ingestion of the meal showed a strong correlation with the plasma concentration of EVs and explained 59–66% of the variation in plasma EVs. These findings suggest that the particle count, measured by NTA, was highly influenced by VLDL particles under postprandial conditions. The reason(s) for the apparent differential impact of VLDL particles on the vesicle count measured by NTA under fasting and postprandial conditions is not known. Even though the plasma concentration of VLDL particles did not increase in postprandial plasma, the mean particle size increased significantly from 42 ± 6 nm in fasting plasma to 55 ± 9 nm in postprandial plasma. With a detection limit of a particle diameter of around 50 nm for EVs by NTA^17^, even a marginal sedimentation of VLDL particles during high-speed centrifugation may affect the particle counts, particularly under postprandial conditions. Thus, our measurements of EVs isolated from plasma by high-speed centrifugation, should be interpreted with caution due to a potential partial interference by VLDL particles, particularly under postprandial conditions. Recently, Mørk *et al*. aimed to prevent VLDL interference during NTA measurements of EVs by antibody-mediated removal of ApoB-exposing lipoproteins from plasma using magnetic beads^33^. However, antibody-mediated depletion of ApoB-containing lipoproteins (including VLDLs) was accompanied by removal of EVs most probably due to the formation of LDL-EV complexes^22^, and by a shift in particle size due to the formation of aggregates of antibodies and lipoproteins^33^. Future studies should optimize centrifugation procedures, antibody-mediated removal or combined procedures in order to separate EVs and VLDL particles, so that NTA can accurately and reliably be applied to measure plasma concentrations of EVs.

In conclusion, isolation of EVs from plasma by high-speed centrifugation yielded similar concentrations and size distributions of EVs for the four anticoagulants tested (citrate, EDTA, CTAD and heparin). We found no statistical difference in concentration nor size of EVs (measured by NTA) when plasma was prepared as PPP or PFP before freezing. Plasma VLDL particles interfered with EV measurements assessed by NTA, particularly under postprandial conditions due to an increase in the median particle diameter of VLDLs exceeding the lower detection limit of NTA. Future studies are warranted to optimize the separation of VLDL particles and EVs in plasma in order to promote the utility of EVs determined by NTA as potential biomarkers of risk, diagnosis and prognosis of diseases.
